# Proteome-Wide Analysis of Disease-Associated SNPs That Show Allele-Specific Transcription Factor Binding

**DOI:** 10.1371/journal.pgen.1002982

**Published:** 2012-09-27

**Authors:** Falk Butter, Lucy Davison, Tar Viturawong, Marion Scheibe, Michiel Vermeulen, John A. Todd, Matthias Mann

**Affiliations:** 1 Department of Proteomics and Signal Transduction, Max Planck Institute of Biochemistry, Martinsried, Germany; 2 Juvenile Diabetes Research Foundation/Wellcome Trust Diabetes and Inflammation Laboratory, Department of Medical Genetics, Cambridge Institute for Medical Research, Addenbrooke's Hospital, University of Cambridge, Cambridge, United Kingdom; 3 Molecular Cancer Research, University Medical Center Utrecht, Utrecht, The Netherlands; Stanford University School of Medicine, United States of America

## Abstract

A causative role for single nucleotide polymorphisms (SNPs) in many genetic disorders has become evident through numerous genome-wide association studies. However, identification of these common causal variants and the molecular mechanisms underlying these associations remains a major challenge. Differential transcription factor binding at a SNP resulting in altered gene expression is one possible mechanism. Here we apply PWAS (“proteome-wide analysis of SNPs”), a methodology based on quantitative mass spectrometry that enables rapid screening of SNPs for differential transcription factor binding, to 12 SNPs that are highly associated with type 1 diabetes at the *IL2RA* locus, encoding the interleukin-2 receptor CD25. We report differential, allele-specific binding of the transcription factors RUNX1, LEF1, CREB, and TFAP4 to *IL2RA* SNPs rs12722508*A, rs12722522*C, rs41295061*A, and rs2104286*A and demonstrate the functional influence of RUNX1 at rs12722508 by reporter gene assay. Thus, PWAS may be able to contribute to our understanding of the molecular consequences of human genetic variability underpinning susceptibility to multi-factorial disease.

## Author Summary

Genome-wide association studies (GWAS) are a powerful approach to identifying genes contributing to risk of disease. However, individual mapped single nucleotide polymorphisms (SNPs) may not map close to a gene, and it can be difficult to distinguish marker SNPs from causal SNPs. Furthermore, the molecular mechanism responsible for disease association is usually not clear. Here we develop a method termed “proteome-wide analysis of SNPs” (PWAS) that identifies differentially binding transcription factors (TFs) and thereby helps to unravel the molecular mechanisms by which the SNPs may exert their effect on gene regulation. We use quantitative interaction proteomics to identify proteins with allele-specific binding. Applied to fine-mapped SNPs conferring risk in type 1 diabetes, PWAS revealed preferential binding of common transcription factors to certain disease-associated SNPs, suggesting they could be causal. In general, a proportion of causal SNPs are likely to function by mimicking binding motifs for transcription factors, increasing their occupancy and modulating gene expression. In addition, PWAS is streamlined and can be used as an informative follow-up approach to GWAS results.

## Introduction

Genome-wide association studies (GWAS) of common diseases typically result in the identification of genomic susceptibility loci, in which several single nucleotide polymorphisms (SNPs) showing strong inter-marker linkage disequilibrium (LD) are equally associated with disease predisposition. Further fine-mapping and re-sequencing studies can then uncover additional SNPs, ideally including those that are causal in the disease etiopathogenesis [1], [2]. However, the SNPs that are most associated with the disease are commonly located in non-coding regions where they have no obvious function. Such SNPs presumably alter expression of a nearby gene via differential transcription factor (TF) binding or by influencing gene splicing. To date, there are few published examples in which a GWAS-identified SNP(s) is correlated with TF binding. We therefore set out to develop an unbiased, sensitive and streamlined method for detection of SNP sequences that differentially bind protein in an allele-specific manner. Affinity purification combined with mass spectrometry (AP-MS) can be a powerful tool to study protein interactions particularly when using a quantitative filter to distinguish specific interactors from the vast majority of background binders by their isotope ratio in the mass spectrometer [3], [4]. The binding of transcription factors to DNA is predominantly mediated by interactions with the phosphate backbone of the DNA. Analysis of differential interactions due to single nucleotide changes is challenging because SNP-related differences in binding affinity are typically low. As a consequence, binding differences are small, even for sequences mutated at multiple positions [5]–[8]. Here we describe PWAS, a technique to study differential transcription factor binding to nucleotide sequences in a streamlined manner. To demonstrate PWAS in a disease relevant context, we also report differential transcription factor binding to type 1 diabetes- (T1D-) associated SNPs at the *IL2RA* or CD25 locus.

## Results

**Figure 1:**
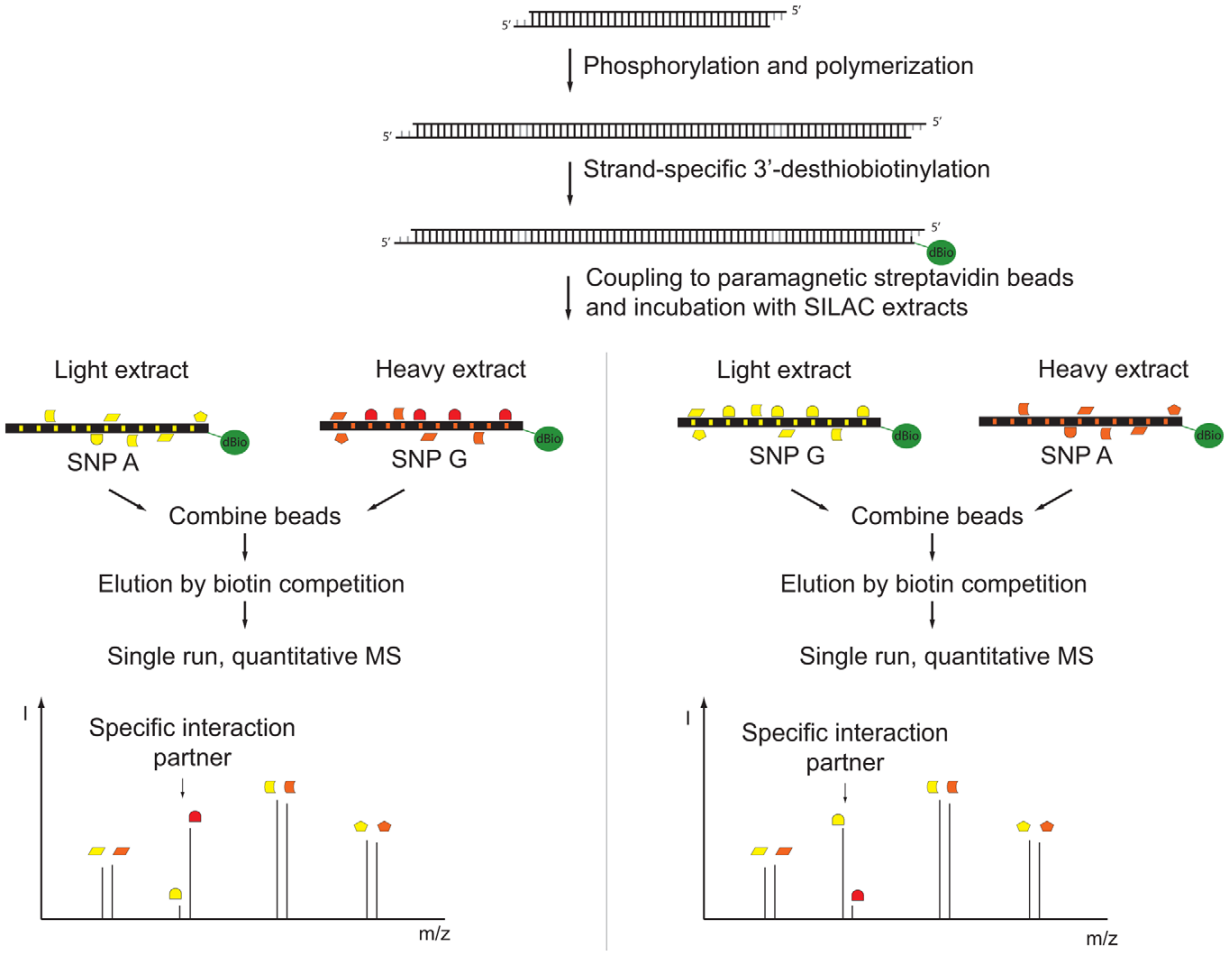
Schematic diagram of SNP pull-down. Synthetic oligonucleotides containing the SNP are phosphorylated, polymerized and subsequently strand-specifically desthiobiotin-labeled. The immobilized DNA fragments are incubated with either light or heavy extract. After removal of unbound proteins, bead fractions are combined and DNA-protein complexes are eluted with biotin. The eluate is precipitated, digested and analyzed by single-run, high resolution, quantitative mass spectrometry. Specific interaction partners result in a ratio different from 1∶1, demonstrating specific enrichment at one variant of the single nucleotide polymorphism.

We improved a recently described technology for DNA affinity capture by quantitative mass spectrometry [5] and developed a pipeline for routine screening of SNPs. To establish an automated protocol for SNP screening, we used a single low stringency buffer for immobilization of the oligonucleotides, incubation with the extracts, and washes. To counterbalance this increased complexity in the lysates, we increased the density of binding sites by concatenation of chemical synthesized DNA oligonucleotides resulting in a greater enrichment of transcription factors. A high enrichment is desirable in mass spectrometric experiments with data-dependent acquisition in order to ensure identification of the desired binding proteins among the majority of peptides originating from background proteins. The chosen TT/AA-overhang further allows incorporation of modified nucleotides by Klenow polymerase. Previously we had used a biotinylated oligonucleotide, which we removed by restriction enzyme cleavage [5], but this introduced a large amount of exogenous protein into the analyzed sample. Here, we performed strand-specific labeling with a desthiobiotin-analog that can be removed conveniently by competition with biotin (Figure 1). The desthiobiotinylated oligonucleotides of the two alleles were then incubated with either light or heavy nuclear extract in parallel. After mild washing, both bead fractions were combined prior to release of the desthiobiotin-labeled oligonucleotide by biotin. We found that PWAS detected differential binding to SNP alleles with great sensitivity. It employs approximately 40 bp of synthetic DNA containing either variant of the SNP, relatively small amounts of nuclear extract (200 µg) that are labeled by SILAC (stable isotope labeling by amino acid in cell culture) [9], [10], single, high resolution mass spectrometric runs, and proved sufficiently simple and robust to be automatable in a robotic format (Figure 1).

**Figure 2:**
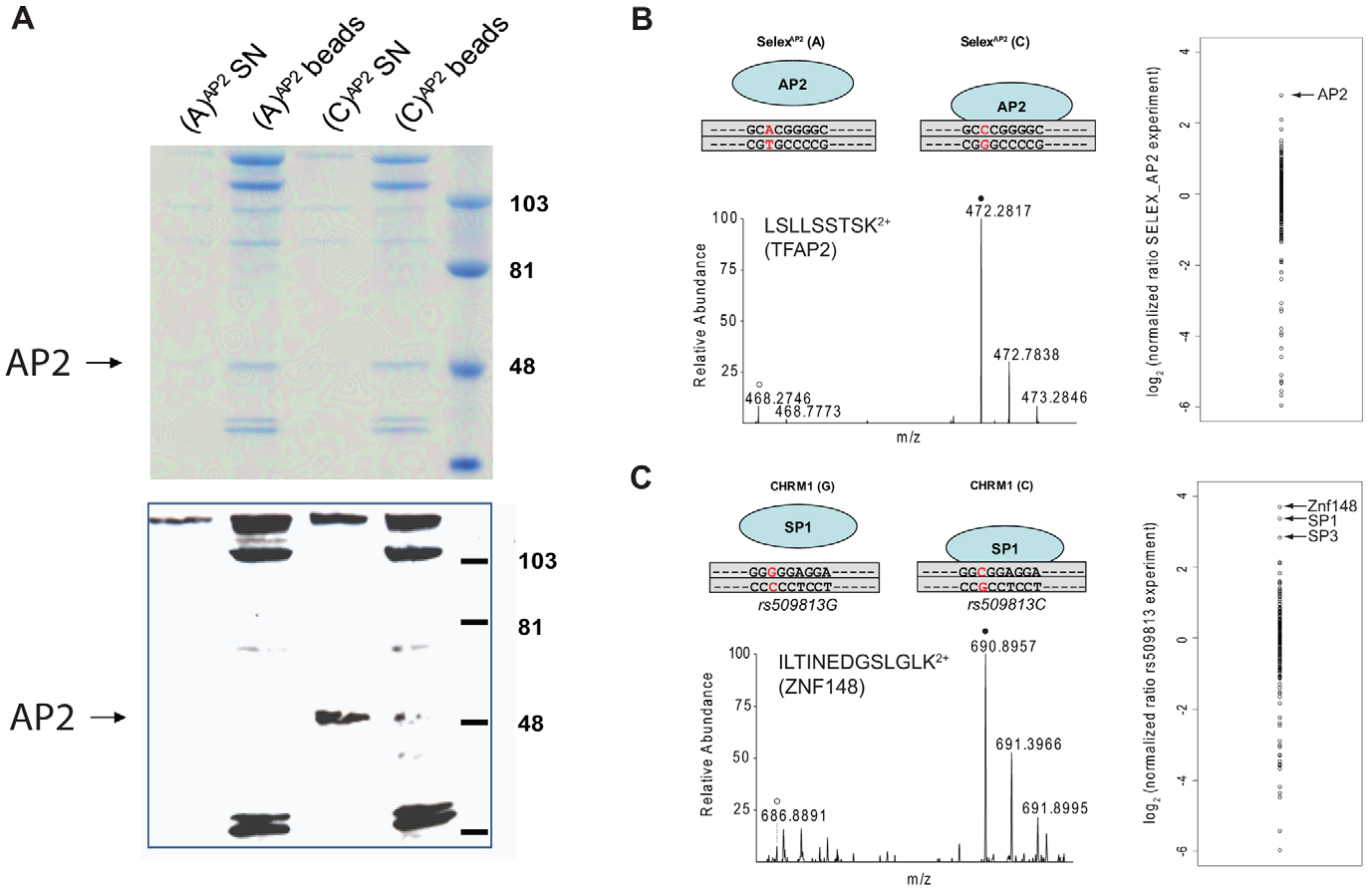
SNP pull-down for TFAP2 and SP1. A: Coomassie stained and anti-TFAP2 immunostained blot. TFAP2 enrichment with the SELEX-derived binding site can be visualized by Western blot, but not within the context of other protein bands in Coomassie staining. B: SNP pull-down for TFAP2. The transcription factor binds to its SELEX sequence (C variant), but a single nucleotide exchange abrogates binding (A variant). Performing proteome-wide detection for differentially binding transcription factors, only TFAP2 is enriched at the wild-type sequence observed in the individual mass spectrum of the TFAP2 peptide LSLLSSTSK^2+^ and by comparison of all SILAC ratios of proteins detected in the experiment. C: SNP pull-down for rs509813. Applying PWAS, we identify SP1 together with SP3, which both bind to the same DNA motif. ZNF148, was also detected as a significant interaction partner in our proteome-wide screen, although not bioinformatically predicted.

We benchmarked our system with a SELEX-derived TFAP2 binding site mutated at a single nucleotide position and a SNP (rs509813 C/G) in the promoter region of the muscarinic acetylcholine receptor M1 (*CHRM1*). This locus is associated with functional differences in gene expression and differential binding of an unidentified transcription factor [11]. While we were only able to visualize TFAP2 binding by immunostaining and not in a Coomassie stained gel (Figure 2A), by mass spectrometry we measured robust and reproducible differential binding of TFAP2 to its SELEX derived binding site with a SILAC ratio of 10 (Figure 2B). For rs509813, we found SP1 as well as SP3 as differential interactors. Both are predicted to bind to the sequence containing the C-allele SNP, but not SP2, which has a slightly different binding motif [12]. Furthermore, we detected binding of the transcription factor ZNF148 (also known as ZBP89) to this region containing rs509813. ZNF148 is a zinc finger protein which has not been predicted to interact with this site, but which has been reported to bind to SP1 binding sites in a mutually exclusive fashion [13] (Figure 2C).

Next, we applied the PWAS methodology to the complex, multi-SNP T1D susceptibility association of the *IL2RA* gene, encoding CD25 in the 10p14 region [1], [2] (Table 1). There are three SNPs in this region which together can be used to tag four common disease-associated haplotypes [1], representing a total of 12 SNPs. The tagging SNPs are rs12722495(A/G), rs11594656(A/T) and rs2104286(A/G). The haplotype (A,A,T) was associated with increased susceptibility to disease, whereas the three haplotypes (G,G,T); (A,A,A) and (A,G,T) were all associated with lower risk of type 1 diabetes. Importantly, these four common haplotypes have also been associated with differences in surface expression of CD25 in T cells, implying that *IL2RA* is a causal gene for T1D in this region [1]. Specifically, individuals with one or two T1D-protective rs12722495 alleles show 27% higher mean CD25 levels on their CD4+ memory T cells compared to fully susceptible individuals or donors with protective rs11594656 or rs2104286 alleles [1]. This is thought to be related to haplotype-dependent transcriptional differences altering CD25 expression, in turn leading to modulation of autoreactivity against pancreatic beta cells. However, it is not known which of the SNPs in the 10p14 region have a direct functional effect, and the identity of the specific transcription factor(s) responsible for this differential binding is equally unclear. Therefore, the precise causal variant(s) in this region has not been determined.

**Table 1:** Overview of all SNPs analyzed in this study with T1D disease association.

SNP	sequence
group 1	
**rs12722495**	CCTTCCAGTTCCTTGAATACTTCCAA[A/G]TCGCACTTAGGATTGAAACTCACCA
rs41295061	TCTGAAGAACCCAGAAGCGACATTAG[A/C]AAGGGGTTCGTTTCACGGAATCCAA
rs12722522	TCGAAGAAAGAGGGCTCATAATTCCA[C/T]GTCAGGGAAGAGCCGCTGGCCTGCC
rs12722508	TGTTGAAAAGAATAGAACCCACCCAC[A/T]GAAACTATCAGAGATCAAATGTTGT
rs41295049	GTGGTGGTATAACATGCAAATGAGAG[A/G]TGCCCAGGGCAAGAAAACTTGCTCT
rs41295065	AGTGGGAGGAAAAGAGAAGAATCAAC[A/G]TGACTCAGATTTCTGGCTTGCGTAC
rs7909519	AGTATAATAGTCAATATAATTAAAAT[G/T]ATTACTTATGCAGTAATTAATTATG
rs41295063	CACCCAGGCTGGAGTGCAACAGTGCA[A/G]TCTCAGCTCACTGCAACTTCTGCCT
group 2	
**rs11594656**	CCAAGGCGGTTCCTTGGTCTGTAGAG[A/T]GAAGGCATCATAGTGAGAAAAGCCA
rs11597367	TACTGGGATTACAAGTGTGAGCCACC[A/G]CACCCAGCGGTTGTGGGCACTCTGA
rs35285258	TTTCTTCTTTTTTAACTTCTATCCCA[C/T]TCATTATACCAAGATCAAACAATAA
group 3	
**rs2104286**	TAGATATAGTCATGGTAACACAAGTC[A/G]TATGTGGTAAGATCTACTGAGCATG

SNPs which mark their respective haplotype are marked in bold.

**Figure 3:**
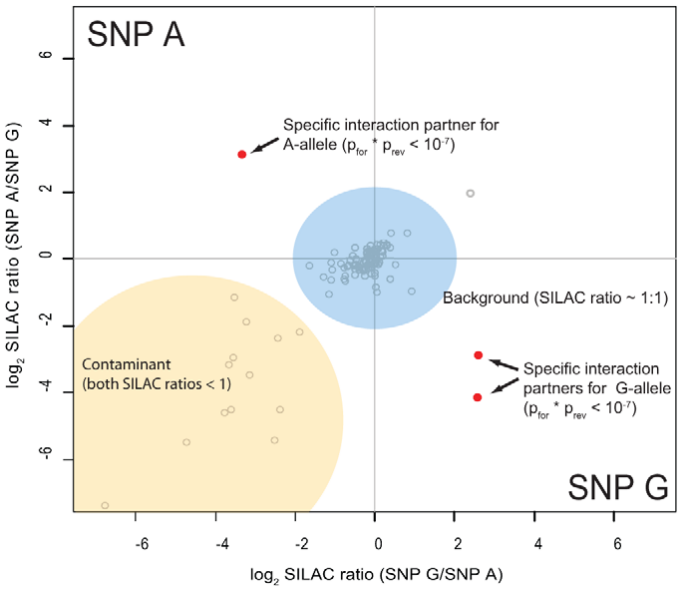
Schematic representation of a two-dimensional interaction plot. While specific outliers are found in the upper left (variant A) or the lower right (variant G) quadrant, most proteins cluster around the origin as they are binding to both variants equally. Contaminants have a SILAC ratios lower than 1 even when labels are switched and thus are grouped in the lower left quadrant.

As preferential binding of transcription factors can occur on either allele, we performed two separate DNA pull-down experiments for each SNP. In the ‘forward’ experiment, the heavy SILAC labeled nuclear extract was incubated with one SNP allele and the light SILAC labeled extract with the other allele. In the ‘reverse’ experiment the SILAC label was switched. As visualized schematically in Figure 3, this strategy allows us to create a two dimensional interaction plot for each pull-down in which interaction partners are grouped into two of four quadrants. Contaminants such as keratins are unlabeled and are sorted into the lower left quadrant because they have a low SILAC ratio in both pull-downs.

We performed 48 SNP pull-downs with the 12 type 1 diabetes SNPs using extracts from the Jurkat T lymphocyte cell line, selected because type 1 diabetes is a T-cell mediated disease (Table S2). A very small number of proteins were clearly separated from the bulk of proteins that bound non-specifically to DNA or to the beads (Figure S1). These outliers were statistically significant in both pull-downs, with a combined forward and reverse pull-down p-value less than 10^−7^. For three SNPs we did not detect significant differential protein binding, suggesting that they may be non-causative and instead represent markers for the causal variant(s).

**Figure 4:**
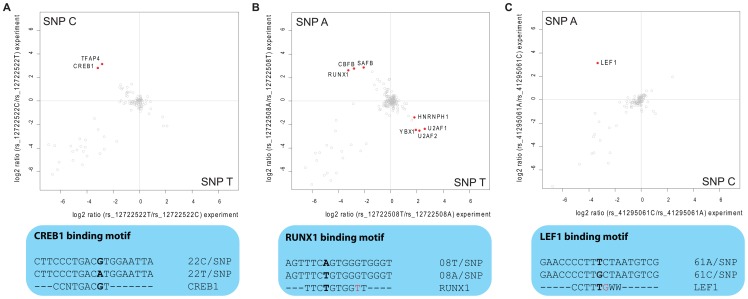
Interaction analysis for fine-mapped T1D SNPs. Two-dimensional interaction plots reveal the specific binding of transcription factors. A: CREB1 and TFAP4 are enriched on rs12722522*C while this allele resembles a perfect match to the CREB consensus binding motif. B: rs12722508 is bound by several transcription factors (upper panel) among them the RUNX1-CBFB heterodimer, although the sequence around rs12722508*A does not match perfectly the transcription factor consensus motif (lower panel); C: Quantitative SNP pull-down screen identifies LEF1 to bind around 8 times stronger to rs41295061*A.

In the eight SNPs of group 1, we found RUNX1 (also known as CBFA) five-fold enriched at allele rs12722508*A (Figure 4B). RUNX1 is a transcriptional regulator likely to be involved in hematopoesis [14], which has already independently been linked to risk of autoimmune disease [15]. Notably, one of the two other significant binders to this SNP is CBFB, which is known to form a heterodimer with RUNX1, underscoring the specificity of our screen [16]. The third differential interactor is SAFB1, a less characterized transcription factor reported to be important in transcriptional regulation of *HSP27* and *ERα* [17]. Additionally, LEF1, a key transcription factor in the Wnt signaling pathway [18] involved in regulating T cell specific genes [19], interacted with rs41295061*A (Figure 4C) in our SNP pull-down experiments. The transcriptional regulators CREB and TFAP4 differentially interacted with rs12722522*C (Figure 4A). The SNP variant rs11597367*G of the three SNP containing group 2 bound ZNF148 and CGGBP1 specifically. We hypothesize that these genotype-dependent interactions are part of the molecular mechanism responsible for the association between these SNPs and expression of CD25 in naïve T cells and stimulated monocytes [1].

Three of the identified transcription factors have known DNA consensus motifs and we therefore investigated whether these motifs were present in the DNA fragment to which they bound. For CREB1 this was indeed the case: the sequence around rs12722522*C ( CGTCA) when reverse complemented reconstituted the binding motif TGACG [20], whereas the other allele was TGACA (Figure 4A). Interestingly, for the other two cases, the region around the SNP did not reconstitute the deposited consensus sequence completely, but generated an additional mismatch (Figure 4B, 4C). However, we note that for RUNX1, the consensus sequence (TGTGGBH) for its murine homologue obtained in a recently published ChIP-seq experiment [21] matches the sequence around rs12722508*A (ACCCACA ) when reverse complemented ( TGTGGGT).

**Figure 5:**
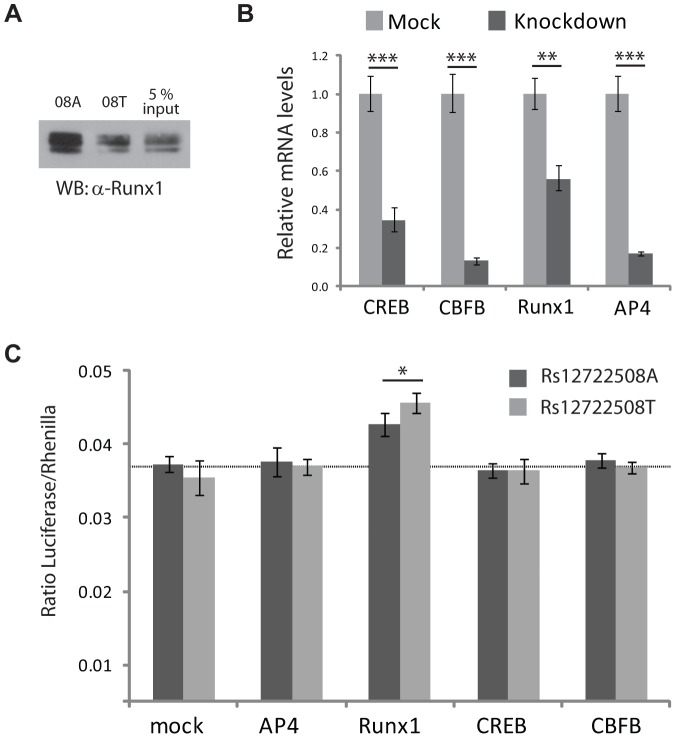
Functional analysis for SNP rs12722508. A: Immunostaining with an antibody against endogenous RUNX1 validates differential binding between the A- and G-allele of rs12722508, input refers to nuclear extract incubated with either rs12722508 allele. B: mRNA levels are reduced upon esiRNA knock-down with mean and s.d. of triplicates; C: changes in firefly luciferase activity in mock-transfection and upon knock-down of TFAP4, RUNX1, CREB and TFAP4. Knock-down of RUNX1 results in an activation with an allele-specific difference (*P* = 0.016) demonstrating the functional consequence of differential RUNX1 binding between the two alleles of rs12722508.

RUNX1 has previously been implicated in other autoimmune diseases [15], which prompted us to further validate this transcription factor - SNP association. We reproduced the allele-specific binding of RUNX1 detected in our mass spectrometric assay by immunostaining (Figure 5A) and investigated the effects on transcription in a transactivation assay. To test whether changes in RUNX1 level would act differently on rs12722508, we reduced the level of this transcription factor by RNA interference (Figure 5B). Upon knock-down of RUNX1, we observed an allele-specific activation of our reporter construct which was not observed when expression levels of control transcription factors were reduced (Figure 5C). Since RUNX1 binds to both alleles, it upregulated both SNP variants; however, consistent with allele-specific differential binding, the upregulation was different between the alleles: rs12722508*A by 16±7 percent and rs12722508*T by 34±11 percent compared to mock (*P* = 0.016). These results link the allele-specific binding of RUNX1 detected in our mass spectrometry screen to functional differences in transcriptional activity.

## Discussion

Our results indicate that differential transcription factor binding to candidate causal SNPs can indicate which SNPs and which TFs might be involved in the causal mechanism(s) from gene-to-protein expression. In the case of SNP group 1 of the *IL2RA* type 1 diabetes locus [1], three of the SNPs differentially bind common transcription factors (rs12722508*A, rs12722522*C and rs41295061*A) implying that there may be more than one SNP within SNP group 1 affecting *IL2RA* transcription. Extrapolating from the SILAC ratios, occupancy of the three SNPs rs12722508*A, rs12722522*C and rs41295061*A was altered between four and eight-fold for these transcription factors. Using reporter assays, we have shown that RUNX1 can mediate allele-specific expression via rs12722508. The relatively small difference measured in our reporter gene assay, and the fact that several SNPs in the haplotype also show differential binding to common TFs, offer a plausible explanation for the observed expression difference of 30% in cell surface CD25 expression [1], [2]. Our results indicate that several sites in a haplotype may contribute to differential transcription factor binding in a cumulative manner, as suggested here for three of eight SNPs of group 1. Supporting such a scenario, genome-wide chromatin immunoprecipitation studies have shown that common transcription factors typically occupy thousands of sites in the genome [22]. We propose that multiple SNPs in a haplotype cooperatively modulate expression levels of nearby genes, contributing to individual traits, including risk of common diseases.

Large scale differential transcription factor binding at SNPs has previously been reported for NFkB and PolII using allele-specific ChIP [23]. Our unbiased, SNP-centered, PWAS approach is orthogonal and complementary to the protein-centered ChIP-seq method and links the detected SNPs from genomics studies directly to the protein without *a priori* knowledge. In conclusion, PWAS is specific, reproducible and generic and only requires synthesis of 40 mers of DNA and batch labeling of cells without the need to obtain cell lines with matching haplotypes. The throughput is currently up to five SNP pairs per day and per mass spectrometer. PWAS may contribute evidence that a given variant is causal. It can thus help to select a subset of polymorphisms from a much larger candidate set that cannot be distinguished by genetic association mapping. Positive results from PWAS directly suggest further gene-phenotype associations that can be investigated to extend the molecular chain of events at least one step further than the GWAS-mapped SNPs themselves.

## Materials and Methods

### Cell culture

Hela S3 and Jurkat cells were SILAC-labeled in RPMI 1640 (-Arg, -Lys) medium containing 10% dialyzed fetal bovine serum (Gibco) supplemented with 84 µg/ml ^13^C_6_^15^N_4_ L-arginine and 40 µg/ml ^13^C_6_^15^N_2_ L-lysine (Sigma Isotec or Cambridge Isotope Labs) or the corresponding non-labeled amino acids, respectively. Nuclear extracts were prepared essentially as described [24].

### Preparation of bait oligonucleotides

25 µg of corresponding pairs of oligonucleotides (Table S1) were annealed and phosphorylated for 2 h at 37°C in the presence of polynucleotide kinase (Fermentas) in 1× T4 ligase buffer. 20 Units T4 ligase (Fermentas) was added to the reaction and incubated at RT overnight. Polymerisation was monitored by agarose gel electrophoresis of a small aliquot of the reaction mixture. After subsequent chloroform-phenol-extraction, the concatemerized oligonucleotides were desthiobiotinylated with d-desthiobiotin-(N6-(6-Amino)hexyl)-dATP (custom synthesis, Jena Biosciences) using 30 units Klenow fragment (Fermentas) overnight at 37°C. Unreacted desthiobiotin nucleotides were removed by size exclusion using a G50 column (GE Healthcare) according to the manufacturer's protocol. The baits were stored at −20°C.

### SNP pulldown

DNA oligonucleotides were immobilized on 50 µl Dynabeads MyOne Streptavidin C1 (Invitrogen) and subsequently incubated with 200 µg of SILAC-labeled nuclear extract (light and heavy separately) in PBB buffer (150 mM NaCl, 50 mM Tris/HCl pH 8.0, 10 mM MgCl_2_, 0.5 percent NP-40, Complete Protease Inhibitor-EDTA [Roche]) for 2 hours at 4°C in a rotation wheel. After three times washing with PBB, bead fractions were pooled and bound DNA-protein complexes were eluted at RT with 200 µl PBB containing 16 mM biotin. The identical steps were automated on a TECAN EVO workstation equipped with a 4 channel LiHAN, robotic arm, cooled buffer storage reservoirs, a temperature controlled shaker and a magnetic separator. Incubations were performed in a 96 well plate (Nunc). Instead of incubation on a rotation wheel, the automated TECAN shaker was operated at 8°C and 700 rpm. Proteins in the elution fraction were precipitated with ethanol and resolubilized in 20 µl 8 M urea for MS analysis.

### LC-MS mass spectrometry

Samples were reduced in 0.5 mM DTT (Sigma) for 30 min, alkylated with 3 mM iodoacetamide (Sigma) for another 30 min and subsequently digested with trypsin (Promega) overnight at room temperature. The digested protein mixture was diluted in 50 mM ammonium bicarbonate buffer pH 8/0.5% TFA and loaded onto a self-made stage tip. MS analysis was performed essentially as previously described [25]. In short, peptides were eluted from stage tips and analyzed by nanoflow liquid chromatography on an EASY-nLC system from Proxeon Biosystems into a LTQ-Orbitrap XL (Thermo Fisher Scientific). Peptides were separated on a C18-reversed phase column packed with Reprosil (Dr. Maisch) directly mounted on the electrospray ion source on an LTQ-Orbitrap XL. We used a 140 min gradient from 2% to 60% acetonitrile in 0.5% acetic acid at a flow of 200 nl/min. The LTQ-Orbitrap XL was operated with a Top5 MS/MS spectra acquisition method in the linear ion trap per MS full scan in the orbitrap.

### Data analysis

The raw files were processed with MaxQuant [26] (version 1.0.12.27) and searched with the Mascot search engine (version 2.2.4.1, Matrix Science) against a IPI human v3.37 protein database concatenated with a decoy of reversed sequences. Carbamidomethylation was set as fixed modification while methionine oxidation and protein N-acetylation were included as variable modifications. The search was performed with an initial mass tolerance of 7 ppm for the precursor ion and 0.5 Da for the MS/MS spectra. Search results were processed with MaxQuant filtered with a false discovery rate of 0.01. Prior to statistical analysis, known contaminants and reverse hits were removed. Proteins identified with at least 1 unique peptide and minimum 2 quantitation events in a single pull-down were considered for analysis and plotted in R (prerelease version 2.8.0).

### Motif analysis

Transcription factors identified for differential SNP binding were checked for deposited recognition sequences in the JASPAR database (www.jaspar.com).

### Immunostaining

For western blotting, proteins were solubilized in LDS sample buffer (Invitrogen), boiled for 5 min at 85°C and fractionated on a 4–12 percent NOVEX gradient gel using MOPS buffer (Invitrogen). Proteins were transferred to a Protran 85 membrane (Whatman) in a blotting chamber (Biorad) at 300 mA for 1 h. The membrane was blocked with PBST (0.1%)+4 percent low fat milk (Roth) for 15 min prior to incubation with the primary antibody for 1 h at room temperature. The following antibodies were diluted in PBST (0.1%) with 4 percent low fat milk: SP1 [1∶1000], TFAP2 [1∶2000], RUNX1 [1∶1000] and LEF1 [1∶1000] (all Abcam). Membrane was washed with PBST (0.1%) three times prior to incubation with either HRP-anti-mouse or HRP-anti-rabbit antibody (both Amersham) for 1 h at room temperature. For detection ECL Western blotting detection reagent (Amersham) was used according to manufacturer's instruction. Chemiluminescence screens (GE Healthcare) were used to visualize the band patterns.

### esiRNA production

Endoribonuclease-prepared short interfering RNAs (esiRNAs) were produced as previously described [27] with the following primers: Rluc (for: GGATAACTGGTCCGCAGTGGT, rev: CCCATTCATCCCATGATTCAA); TFAP4 (for: GTGCCCTCTTTGCAACATTT, rev: TTCTCGTCCTCCCAGATGTC); RUNX1 (for: GGCTGGCAATGATGAAAACT, rev: GATGGTTGGATCTGCCTTGT); CREB1 (for: GGAGTGCCAAGGATTGAAGA, rev: CCTCTCTCTTTCGTGCTGCT); CBFB (for: TTTGAAGGCTCCCATGATTC, rev: CCATGGCAGTTTGTGATGTC).

### Firefly reporter constructs

The heat shock promoter was amplified (Hsp68_for: GAGAAAGCTTCAGGAACATCCAAACTGAGCA, Hsp68_rev: GAGAAAGCTTCGCTTGTCTCTGGATGGAAC) from genomic DNA prepared from C2C12 cells. The promoter was cloned into pGL3-basic (Promega) in front of the firefly luciferase gene at the HindIII site. A gateway cassette was inserted 5′ of the promoter to facilitate insertion of different DNA sequences, generating the pGL3/GW/mHsp68prom vector. A triple repeat of the sequence surrounding rs12722508 (AACCCACCCAC[A/T]GAAACTATCAGAG) was cloned into pCR8 and transferred into pGL3/GW/mHsp69prom using LR recombination.

### Reporter assay

100,000 cells HeLa Kyoto were reverse transfected with 150 ng esiRNA and 1 µl oligofectamine (Invitrogen) in complete medium in a 24 well plate. After 16 hours, 4 ng Rhenilla luciferase vector phRL-TK (Promega) were co-transfected with 200 ng firefly reporter (pGL3/GW3/mHsp69prom) containing either SNP variant with Optimem medium (Gibco). Medium was exchanged against complete medium 4 hours past transfection. Cells were harvest the next day using passive lysis buffer (Promega). Independent biological triplicates were measured on the GloMax luminometer (Promega) using the Dual Luciferase Assay kit (Promega) according to the manufacturer's protocol. For representation, the mean and the standard deviation were calculated. Transfection efficiency was monitored by parallel transfection of RLuc esiRNA targeting rhenilla luciferase.

### qRT–PCR

Cells form one well (24 well plate) transfected with esiRNA and reporter assay constructs were trypsinized, washed in PBS and RNA extracted using a spin filter kit (UBS). Around 1 µg of pure RNA were reverse-transcribed with polyT primers using the Fast cDNA kit (Fermentas). The following primers were used for the amplification: TFAP4 (for: GCAGACAGCCGAGTACATCTT, rev: CCTATGCCTTCGTCCTTGTCC), RUNX1 (for: GATGGCACTCTGGTCACTGTGA, rev: CTTCATGGCTGCGGTAGCAT), CBFB (for: AGAAGCAAGTTCGAGAACGAG, rev: GAAGCCCGTGTACTTAATCTCAC), CREB (for: CACCTGCCATCACCACTGTAA, rev: GCTGCATTGGTCATGGTTAATGT), GAPDH (for: TGCACCACCAACTGCTTAGC, rev: GGCATGGACTGTGGTCATGAG). Three independent replicates using the IQ SybrGreen supermix (Biorad) were measured on a CFX96 qRT-PCR machine (Biorad). For assessment of knock-down the *ΔΔC_t_* method was used.

## Supporting Information

Figure S1 SNP pull-downs were performed in duplicates switching the labels for each of the SNPs. Group 1: rs12722522, rs12722508, rs12722495, rs41295061 (alias ss52580101), rs41295049 (alias ss52580073) and rs41295065 (alias ss52580109), rs41295063 and rs7909519; Group 2: rs11597367, rs11594656 and rs35285258 (alias ss52580135); Group 3: rs2104286. Two-dimensional interaction plots showing enrichment of transcription factors with one variant of the SNP by sorting them in the lower right or upper left quadrant, having inversed high ratios in both pull-downs. Contaminants can be visualized in the lower left quadrant having a low ratio in both experiments.
(PDF)

Table S1 Primers used in 5′-3′ direction.
(PDF)

Table S2 Protein identifications from PWAS screen for *IL2RA* SNPs.
(XLSX)
