# Supplementary figures and images for: Proteome-Wide Analysis of Disease-Associated SNPs That Show Allele-Specific Transcription Factor Binding
Source: PLoS Genet. 2012 Sep 27;8(9):e1002982. doi: 10.1371/journal.pgen.1002982 (PMC3459973; doi:10.1371/journal.pgen.1002982)

Figure S 1

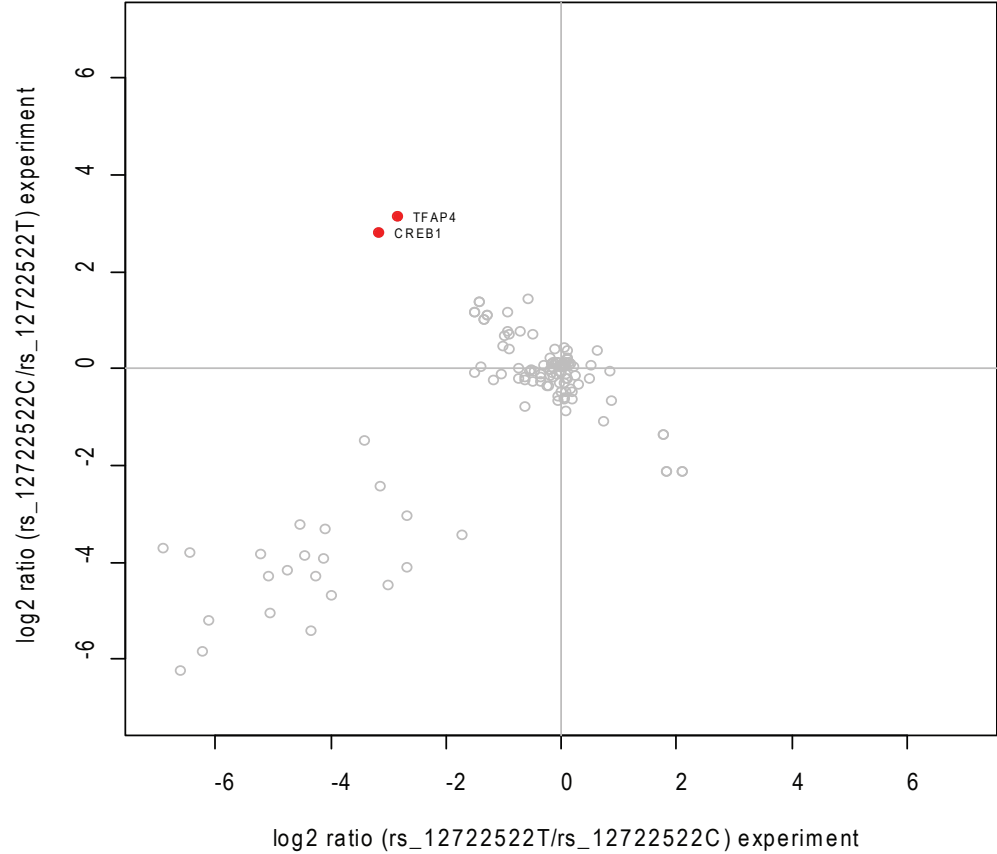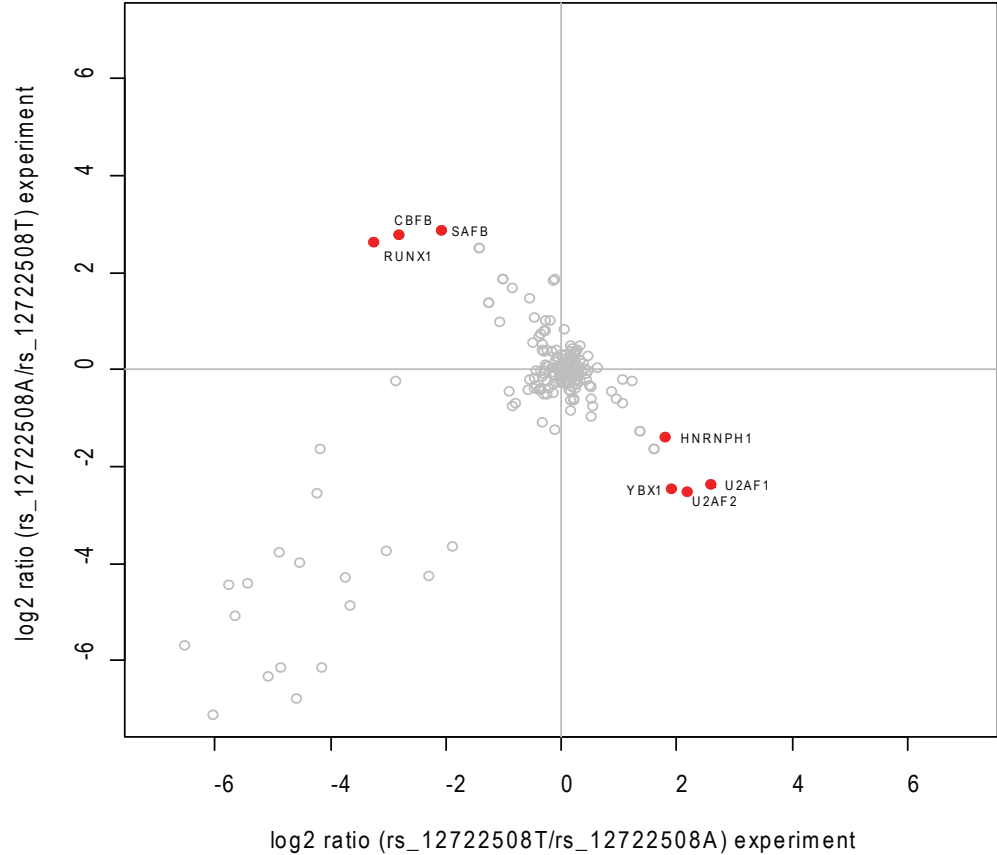

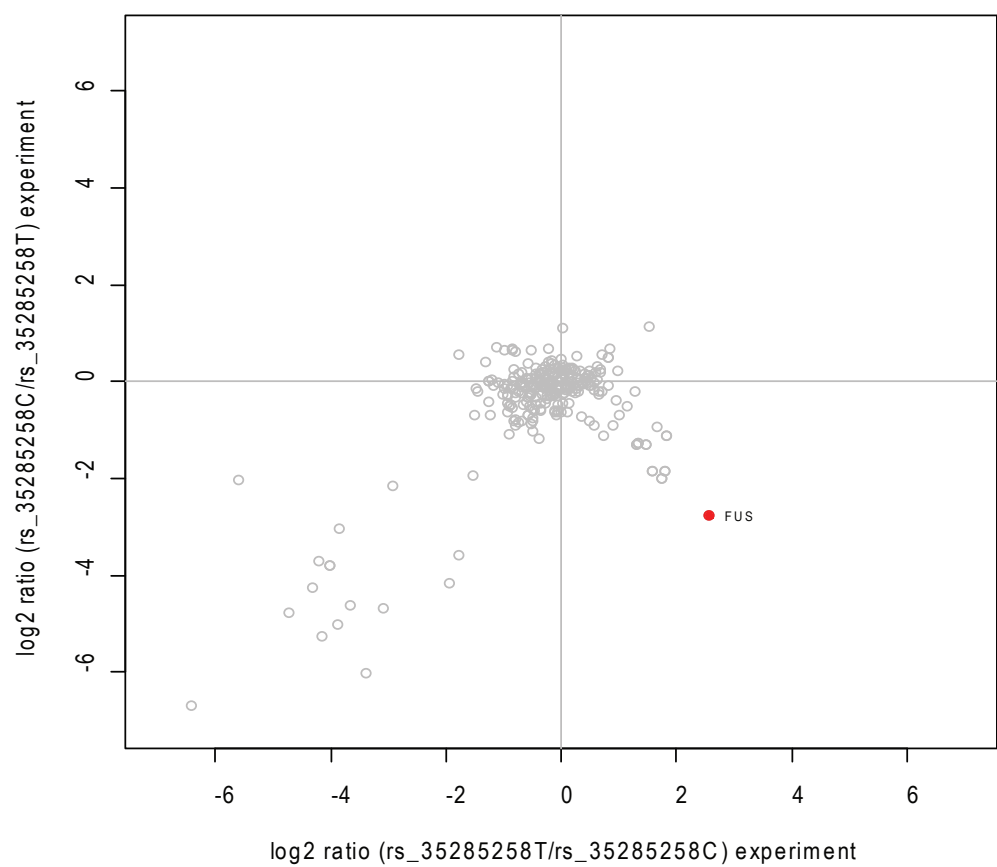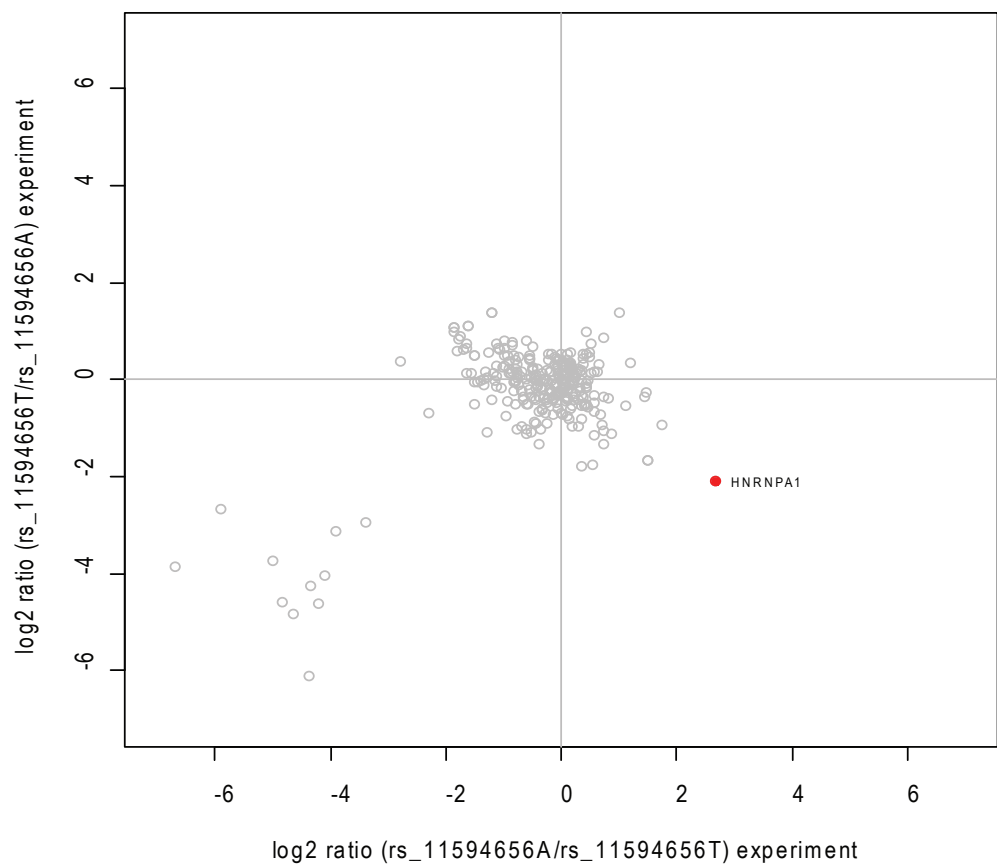

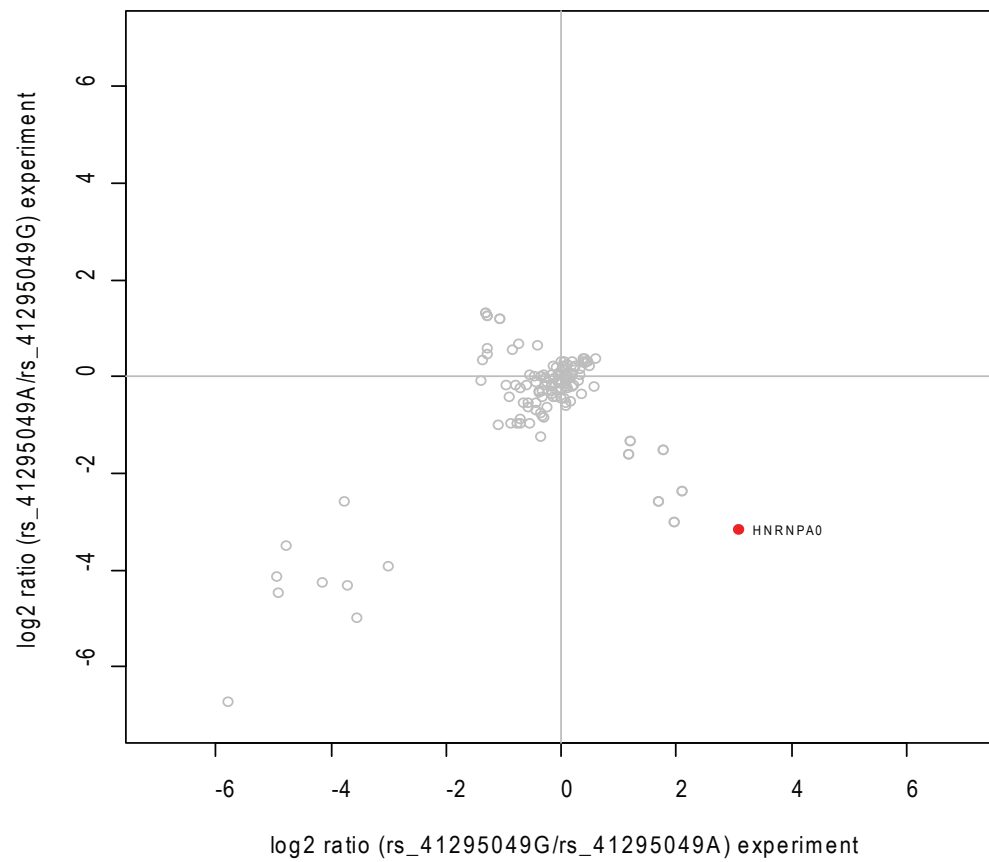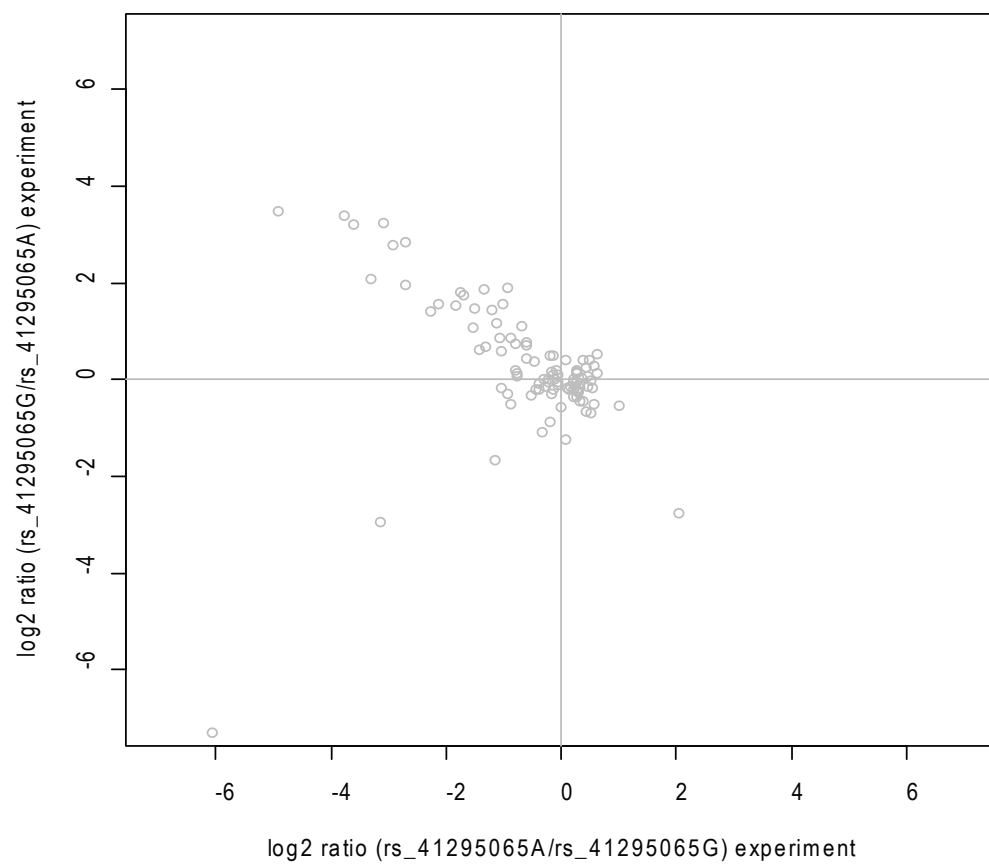

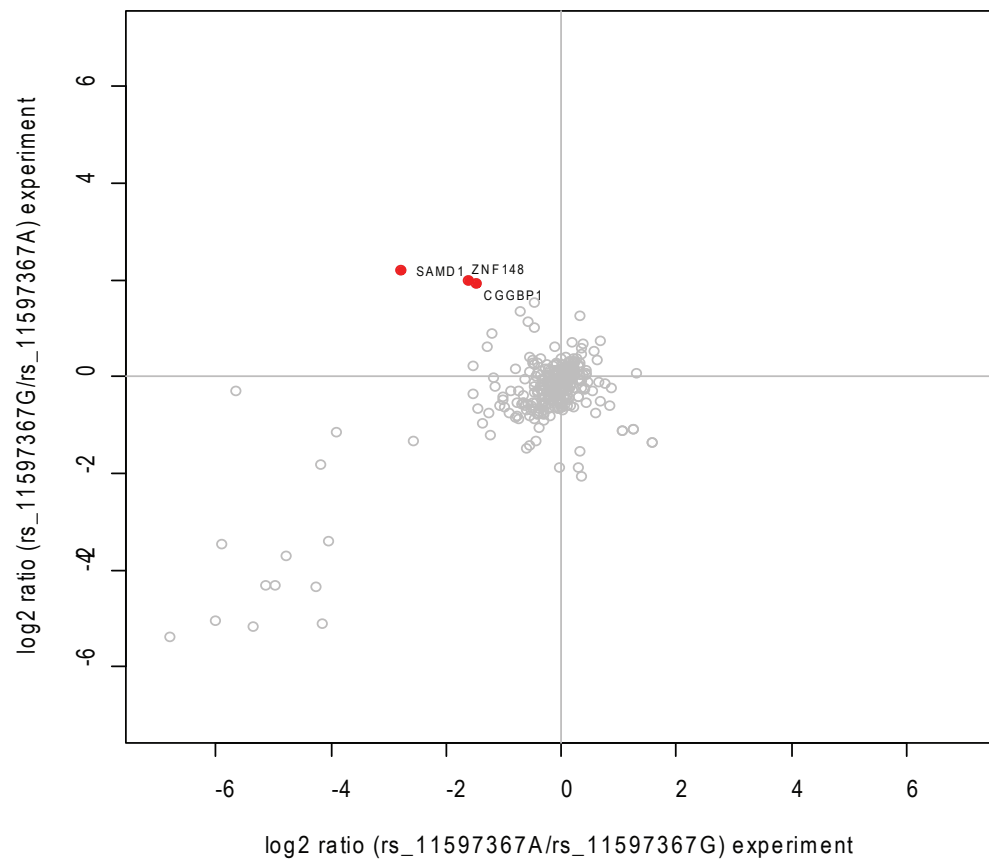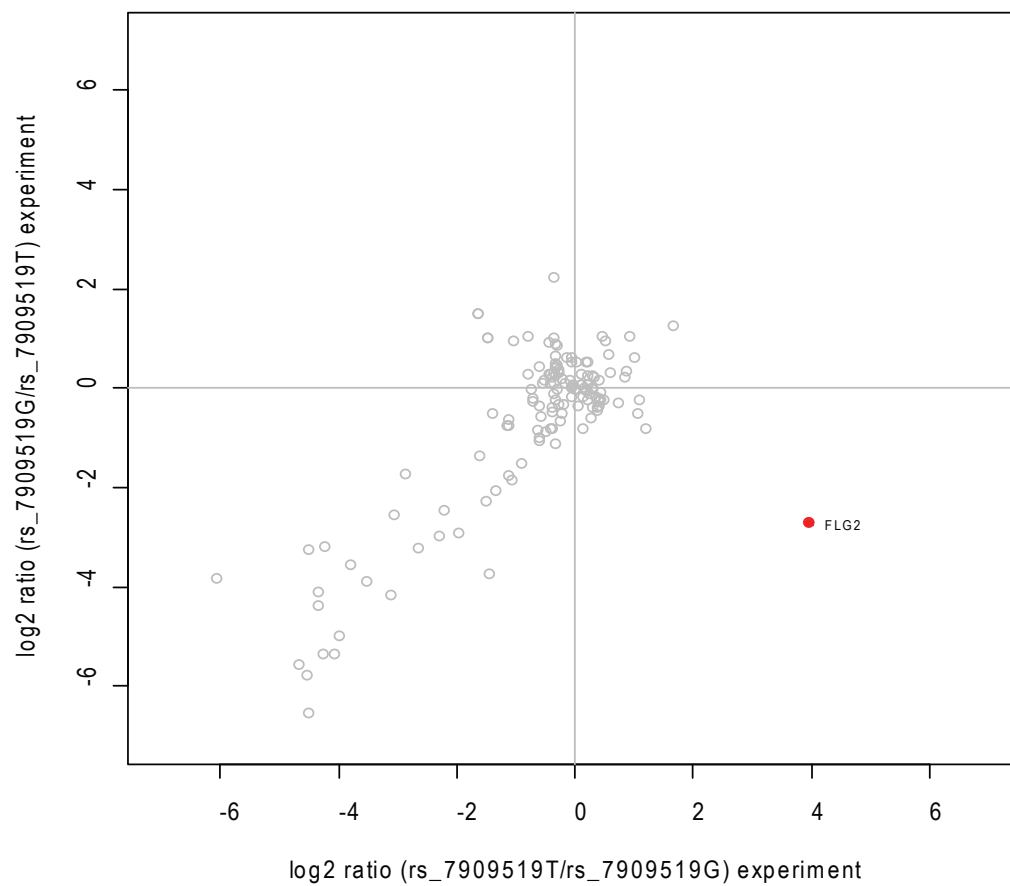

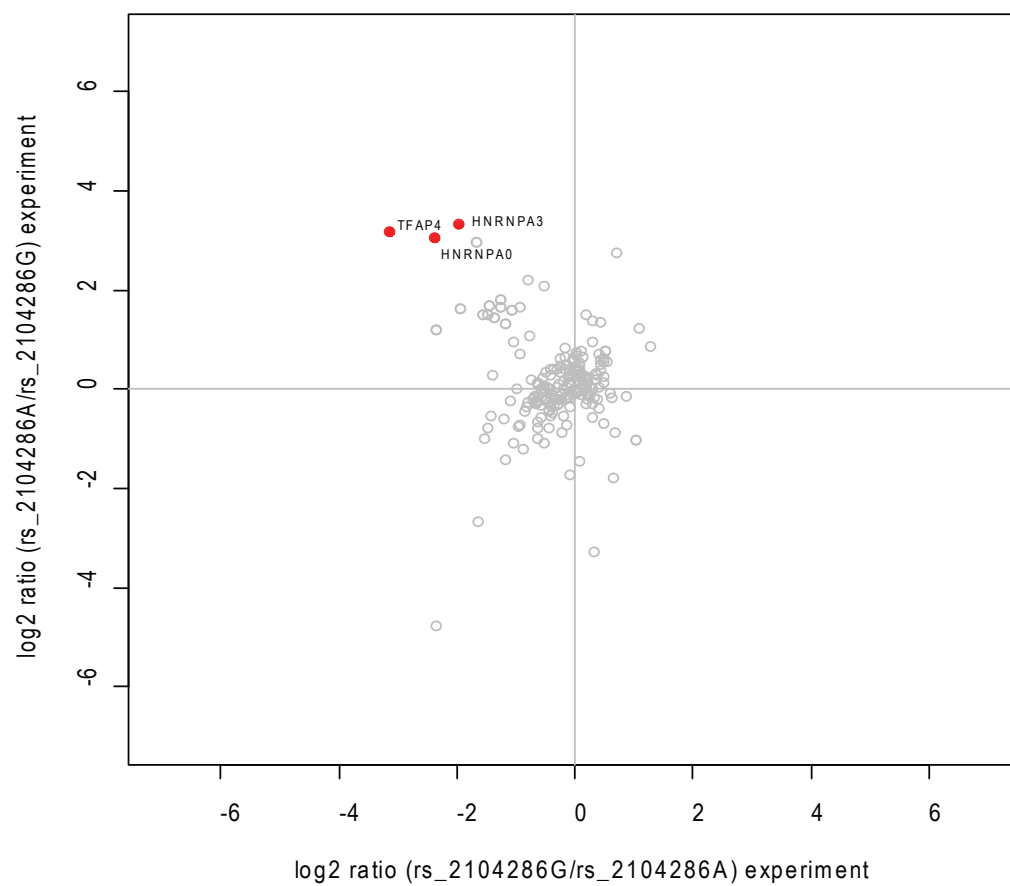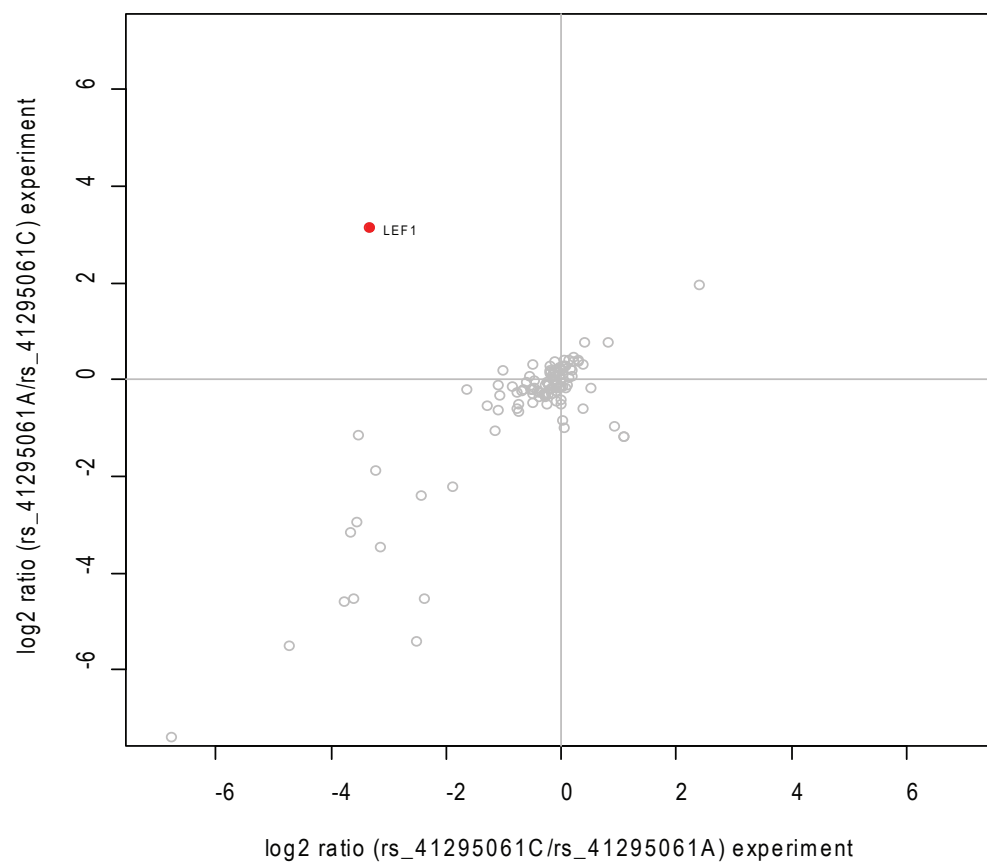

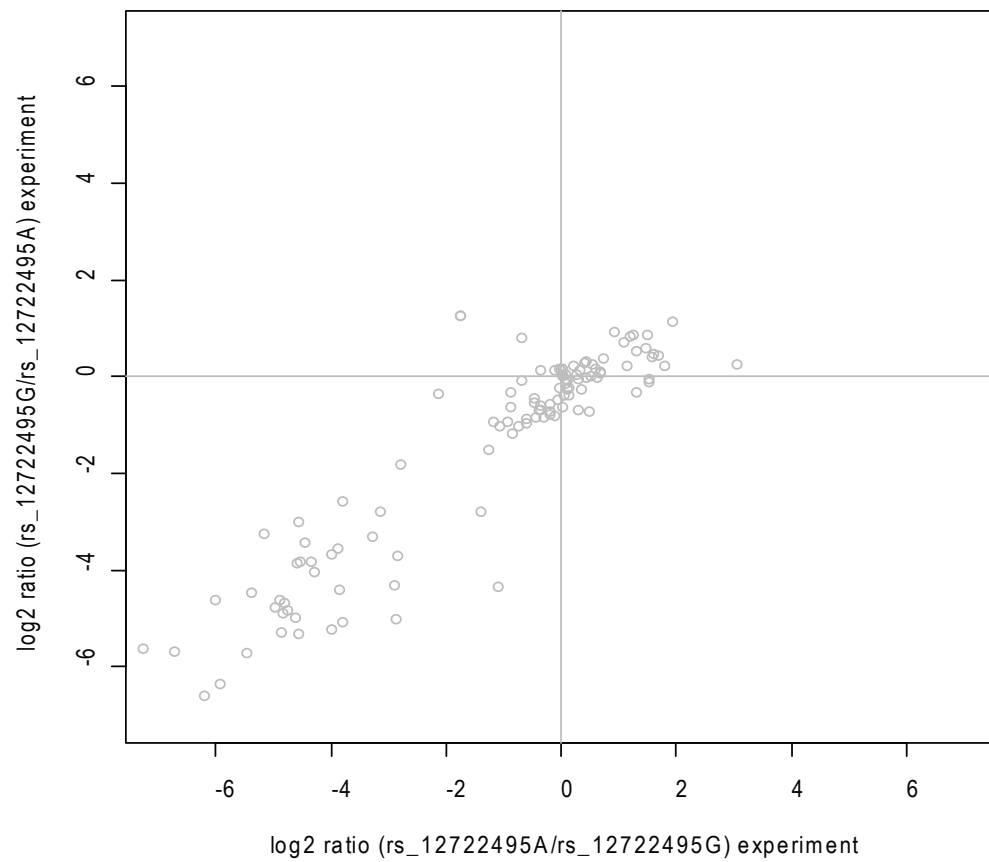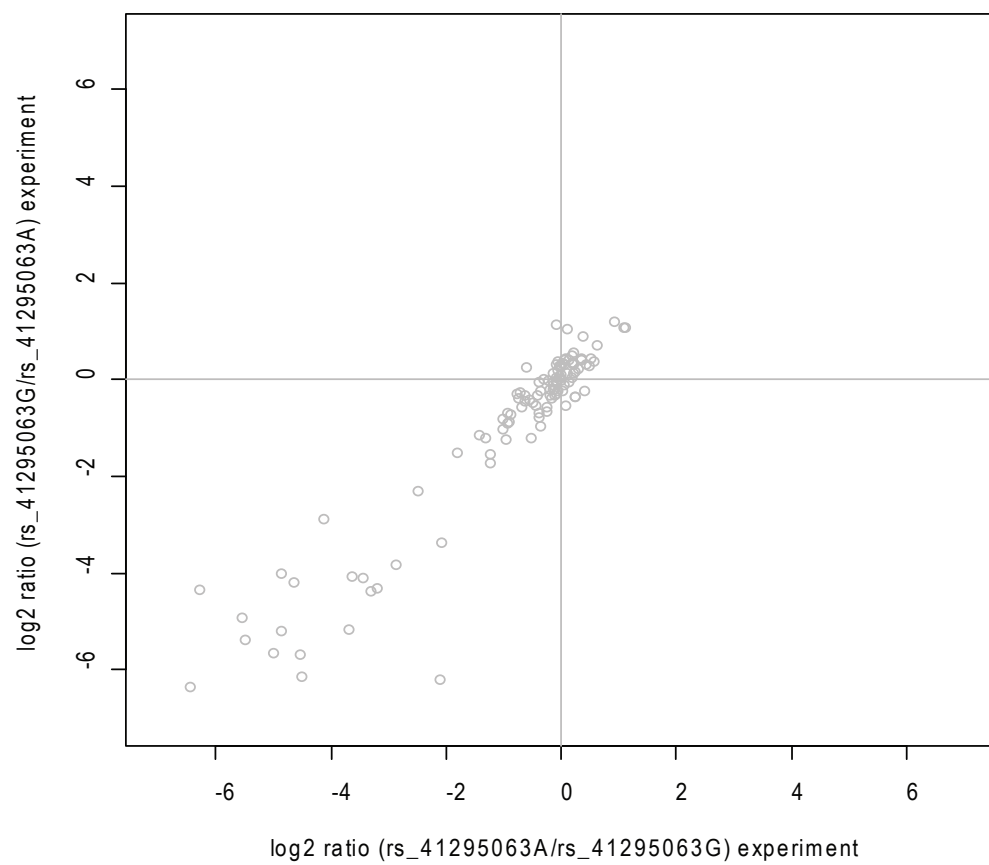

Supplement: Figure S1 — SNP pull-downs were performed in duplicates switching the labels for each of the SNPs. Group 1: rs12722522, rs12722508, rs12722495, rs41295061 (alias ss52580101), rs41295049 (alias ss52580073) and rs41295065 (alias ss52580109), rs41295063 and rs7909519; Group 2: rs11597367, rs11594656 and rs35285258 (alias ss52580135); Group 3: rs2104286. Two-dimensional interaction plots showing enrichment of transcription factors with one variant of the SNP by sorting them in the lower right or upper left quadrant, having inversed high ratios in both pull-downs. Contaminants can be visualized in the lower left quadrant having a low ratio in both experiments. (PDF) [file pgen.1002982.s001.pdf]
