# Supplementary material for: Proteome-Wide Analysis of Disease-Associated SNPs That Show Allele-Specific Transcription Factor Binding
Source: PLoS Genet. 2012 Sep 27;8(9):e1002982. doi: 10.1371/journal.pgen.1002982 (PMC3459973; doi:10.1371/journal.pgen.1002982)
Supplement: Table S1 — Primers used in 5′-3′ direction. (PDF) [file pgen.1002982.s002.pdf]

Table S1

**AP2_C_for**	AACAAAACTGCAGGATCGAACTGACCGCCCGGGGCACGTGTC
**AP2_C_rev**	TTGACACGTGCCCCGGGCGGTCAGTTCGATCCTGCAGTTTTG
**AP2_A_for**	AACAAAACTGCAGGATCGAACTGACCGCACGGGGCACGTGTC
**AP2_A_rev**	TTGACACGTGCCCCGTGCGGTCAGTTCGATCCTGCAGTTTTG
**rs509813C_for**	AAGGCTTGGGCTCCTCCCCCCAGCCAAC
**rs509813C_rev**	TTGTTGGCTGGGGGGAGGAGCCCAAGCC
**rs509813G_for**	AAGGCTTGGGCTCCTCCCGCCAGCCAAC
**rs509813G_rev**	TTGTTGGCTGGCGGGAGGAGCCCAAGCC
**rs2104286A_for**	TTAGTCATGGTAACACAAGTCATATGTGGTAAGATCTAC
**rs2104286A_rev**	AAGTAGATCTTACCACATATGACTTGTGTTACCATGACT
**rs2104286G_for**	TTAGTCATGGTAACACAAGTCGTATGTGGTAAGATCTAC
**rs2104286G_rev**	AAGTAGATCTTACCACATACGACTTGTGTTACCATGACT
**rs11594656A_for**	TTCGGTTCCTTGGTCTGTAGAGAGAAGGCATCATAGTGAG
**rs11594656A_rev**	AACTCACTATGATGCCTTCTCTCTACAGACCAAGGAACCG
**rs11594656T_for**	TTCGGTTCCTTGGTCTGTAGAGTGAAGGCATCATAGTGAG
**rs11594656T_rev**	AACTCACTATGATGCCTTCACTCTACAGACCAAGGAACCG
**rs41295061A_for**	TTGAACCCAGAAGCGACATTAGAAAGGGGTTCGTTTCACGGAATC
**rs41295061A_rev**	AAGATTCCGTGAAACGAACCCCTTTCTAATGTCGCTTCTGGGTTC
**rs41295061C_for**	TTGAACCCAGAAGCGACATTAGCAAGGGGTTCGTTTCACGGAATC
**rs41295061C_rev**	AAGATTCCGTGAAACGAACCCCTTGCTAATGTCGCTTCTGGGTTC
**rs12722522C_for**	TTGAAAGAGGGCTCATAATTCCACGTCAGGGAAGAGCCGCTGGC
**rs12722522C_rev**	AAGCCAGCGGCTCTTCCCTGACGTGGAATTATGAGCCCTCTTTC
**rs12722522T_for**	TTGAAAGAGGGCTCATAATTCCATGTCAGGGAAGAGCCGCTGGC
**rs12722522T_rev**	AAGCCAGCGGCTCTTCCCTGACATGGAATTATGAGCCCTCTTTC
**rs12722508A_for**	TTGAAAAGAATAGAACCCACCCACAGAAACTATCAGAGATCAAATG
**rs12722508A_rev**	AACATTTGATCTCTGATAGTTTCTGTGGGTGGGTTCTATTCTTTTC
**rs12722508T_for**	TTGAAAAGAATAGAACCCACCCACTGAAACTATCAGAGATCAAATG
**rs12722508T_rev**	AACATTTGATCTCTGATAGTTTCAGTGGGTGGGTTCTATTCTTTTC
**rs12722495A_for**	TTCCAGTTCCTTGAATACTTCCAAATCGCACTTAGGATTGAAAC
**rs12722495A_rev**	AAGTTTCAATCCTAAGTGCGATTTGGAAGTATTCAAGGAACTGG
**rs12722495G_for**	TTCCAGTTCCTTGAATACTTCCAAGTCGCACTTAGGATTGAAAC
**rs12722495G_rev**	AAGTTTCAATCCTAAGTGCGACTTGGAAGTATTCAAGGAACTGG
**rs41295049A_for**	TTGGTATAACATGCAAATGAGAGATGCCCAGGGCAAGAAAACTTG
**rs41295049A_rev**	AACAAGTTTTCTTGCCCTGGGCATCTCTCATTTGCATGTTATACC
**rs41295049G_for**	TTGGTATAACATGCAAATGAGAGGTGCCCAGGGCAAGAAAACTTG
**rs41295049G_rev**	AACAAGTTTTCTTGCCCTGGGCACCTCTCATTTGCATGTTATACC
**rs41295065A_for**	TTGGAGGAAAAGAGAAGAATCAACATGACTCAGATTTCTGGCTTG
**rs41295065A_rev**	AACAAGCCAGAAATCTGAGTCATGTTGATTCTTCTCTTTTCCTCC
**rs41295065G_for**	TTGGAGGAAAAGAGAAGAATCAACGTGACTCAGATTTCTGGCTTG
**rs41295065G_rev**	AACAAGCCAGAAATCTGAGTCACGTTGATTCTTCTCTTTTCCTCC
**rs41295063A_for**	TTGCTGGAGTGCAACAGTGCAATCTCAGCTCACTGCAACTTC
**rs41295063A_rev**	AAGAAGTTGCAGTGAGCTGAGATTGCACTGTTGCACTCCAGC
**rs41295063G_for**	TTGCTGGAGTGCAACAGTGCAGTCTCAGCTCACTGCAACTTC
**rs41295063G_rev**	AAGAAGTTGCAGTGAGCTGAGACTGCACTGTTGCACTCCAGC
**rs7909519G_for**	TTAATAGTCAATATAATTAAAATGATTACTTATGCAGTAATTA
**rs7909519G_rev**	AATAATTACTGCATAAGTAATCATTTTAATTATATTGACTATT
**rs7909519T_for**	TTAATAGTCAATATAATTAAAATTATTACTTATGCAGTAATTA
**rs7909519T_rev**	AATAATTACTGCATAAGTAATAATTTTAATTATATTGACTATT
**rs11597367A_for**	TTGGATTACAAGTGTGAGCCACCACACCCAGCGGTTGTGGGCA
**rs11597367A_rev**	AATGCCCACAACCGCTGGGTGTGGTGGCTCACACTTGTAATCC
**rs11597367G_for**	TTGGATTACAAGTGTGAGCCACCGCACCCAGCGGTTGTGGGCA
**rs11597367G_rev**	AATGCCCACAACCGCTGGGTGCGGTGGCTCACACTTGTAATCC
**rs35285258C_for**	TTCTTTTTTAACTTCTATCCCACTCATTATACCAAGATCAAA
**rs35285258C_rev**	AATTTGATCTTGGTATAATGAGTGGGATAGAAGTTAAAAAAG
**rs35285258T_for**	TTCTTTTTTAACTTCTATCCCATTCATTATACCAAGATCAAA
**rs35285258T_rev**	AATTTGATCTTGGTATAATGAATGGGATAGAAGTTAAAAAAG
